# Physical Properties of Molecules and Condensed Materials Governed by Onsite Repulsion, Spin-Orbit Coupling and Polarizability of Their Constituent Atoms

**DOI:** 10.3390/molecules25040867

**Published:** 2020-02-16

**Authors:** Paul A. Maggard, Xiyue Cheng, Shuiquan Deng, Myung-Hwan Whangbo

**Affiliations:** 1 Department of Chemistry, North Carolina State University, Raleigh, NC 27695-8204, USA; 2 State Key Laboratory of Structural Chemistry, Fujian Institute of Research on the Structure of Matter, Chinese Academy of Sciences, Fuzhou 350002, China; xycheng@fjirsm.ac.cn

**Keywords:** onsite repulsion, spin–orbit coupling, polarizability, Aufbau principle, periodic table

## Abstract

The onsite repulsion, spin–orbit coupling and polarizability of elements and their ions play important roles in controlling the physical properties of molecules and condensed materials. In celebration of the 150th birthday of the periodic table this year, we briefly review how these parameters affect the physical properties and are interrelated.

## 1. Introduction

Mendeleev’s periodic table of elements, which was formulated in 1869 based on atomic weights, paved the way for its reformulation in terms of atomic numbers [1]. Subsequently, the occurrence of the periods and groups in the periodic table were explained in the early 1920s by Bohr and Pauli, who formulated the Aufbau (build-up) principle based on the shell structure of atoms discovered from the newly developed quantum theory of atoms [1]. This principle predicts the ground state electron configurations of atoms when supplemented by Hund’s rule (i.e., if degenerate levels are available, electrons will occupy different orbitals singly before any are occupied doubly) and the Pauli principle (i.e., electrons occupying the same orbital must have different spins) [1]. In the last 150 years, the trends in the properties of the elements (electronegativity, ionization potential, electron affinity, atomic size, spin–orbit coupling (SOC), polarizability, etc.) across each period and within each group of the periodic table have been indispensable in understanding a vast number of physical and chemical properties of discrete molecules and condensed materials. 

Although all elements of a given group exhibit similar behaviors in their chemical bonding, they show subtle differences in their properties, primarily because their atomic sizes increase as one moves down a period. As the size of an atom increases, the valence orbitals increase their radial extensions, thereby increasing their diffuseness. The latter reduces not only the degree of electron–electron repulsion among its valence electrons (i.e., the onsite repulsion U), but also makes the valence electrons more polarizable to the oscillating electric field of a light. An element of a given group can adopt the behaviors of its neighboring groups when it becomes a cation by losing electrons or an anion by gaining electrons. An important consequence of these ionizations is that an element becomes smaller in size when it becomes a cation, but larger when it becomes an anion. Due to this change in size, the onsite repulsion, SOC and polarizability of the ion become different from those of its neutral analogue. 

In celebration of the 150th birthday of the periodic table this year, we briefly review how the physical properties of molecules and condensed materials are controlled by the onsite repulsion, SOC and polarizability of elements and their ions. Our work is organized as follows: we show that, in most cases, the consideration of onsite repulsion can correct the deficiency of the Aufbau principle for molecules in Section 2, and for condensed materials in Section 3. Important roles of SOC and polarizability in controlling physical properties are discussed in Section 4 and Section 5, respectively. We examine how the onsite repulsions, SOC and polarizabilities of transition metal cations are interrelated in Section 6, and summarize our concluding remarks in Section 7.

## 2. Inadequacy of the Aufbau Principle 

To accurately describe the ground-state of a discrete molecule, it is necessary to carry out first principles calculations using a determinant wavefunction, which is antisymmetric as required by quantum mechanics. A determinant wavefunction is constructed using molecular orbitals (MOs) expressed as a linear combination of atomic orbitals (AOs). The energies and AO compositions of the occupied MOs are repeatedly refined from an initial guess until an additional cycle of calculations does not lower the total energy any further. The inspection of the occupied MOs resulting from such self-consistent-field (SCF) calculations enables one to find the electron configuration describing the ground state. The total energy of a system depends on the energies of the occupied MOs and also on the Coulomb and exchange repulsions between the occupied MOs. To be quantitatively more accurate, one may go beyond the level of one-determinant wavefunction description by carrying out, for example, extensive configuration interaction (CI) calculations. 

Though quantitatively accurate, such calculations based on first principles do not provide a conceptual tool with which to speculate the possible outcomes of SCF calculations and/or experiments designed to test the consequences of possible ground-state electron configurations. For such qualitative discussions, it is convenient to employ the MO levels generated by the one-electron theory, because the relative energies of various electron configurations are determined solely by the relative energies of MOs. Obviously, being qualitative, the one-electron approach can lead to erroneous conclusions. In cases when incorrect predictions result from the Aufbau principle coupled with the Hund’s rule and the Pauli principle, hereafter referred to as the Aufbau principle, one can correct them in most cases by introducing the concept of onsite repulsion.

### 2.1. Low-Spin vs. High-Spin States

**Figure 1:**
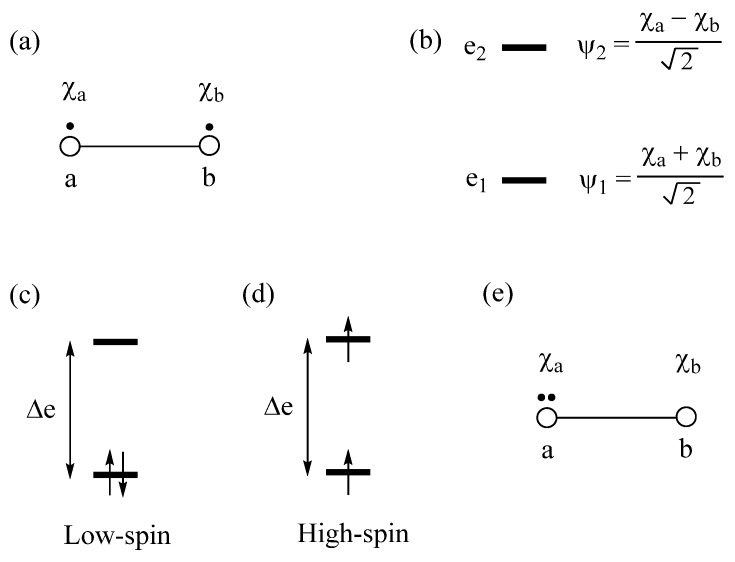
(**a**) A dimer made up of two identical atoms. The AOs at the sites a and b are labeled as χ_a_ and χ_b_, respectively. (**b**) The bonding and antibonding MOs, ψ_1_ and ψ_2_, respectively, separated by energy ∆e. (**c**) The low-spin state of a dimer. (**d**) The high-spin state of a dimer. (**e**) The double occupation of one AO leading to the on-site repulsion U.

Exchange repulsions between the MOs occupied with identical spins arise from the requirement that a wavefunction be antisymmetric, affecting the predictions of the Aufbau principle. We illustrate this point by considering a dimer made up of two identical atoms, each with one AO and one electron (Figure 1a). The interaction between the two AOs, χ_a_ and χ_b_, located at sites *a* and *b*, respectively, gives rise to the bonding molecular orbital (MO) ψ_1_ and the antibonding MO ψ_2_ (Figure 1b), separated by an energy ∆e. If *t* is the interaction energy (i.e., the hopping integral) between the orbitals χ_a_ and χ_b_, then ∆e ≈ 2|*t*|. As long as ∆e is nonzero, the Aufbau principle predicts that the ground state is the low-spin (LS) state in which the bonding MO ψ_1_ is doubly occupied (Figure 1c), and that the high-spin (HS) state in which the bonding and antibonding MOs are each singly occupied with identical spin (Figure 1d) is the excited state. In terms of the orbital energy, the LS state is more stable than the HS state by ∆e. However, the two states differ in their electron–electron repulsions. The LS state has the Coulomb repulsion J_11_, while the HS state has the Coulomb repulsion J_12_ and the exchange repulsion –K_12_. In terms of the onsite repulsion U, namely, the electron–electron repulsion arising from the double-occupancy of one AO (Figure 1e), it is found that J_11_ ≈ U/2 and J_12_ – K_12_ ≈ 0. In terms of onsite repulsion, therefore, the LS state is less stable than the HS state by U/2. Consequently, the LS state is more stable than the HS state if ∆e − U/2 > 0, but the opposite is the case if ∆e − U/2 < 0 [2]. In short, the shortcoming of the Aufbau principle is corrected by introducing the concept of onsite repulsion. 

### 2.2. Extension of Hund’s Rule

**Figure 2:**
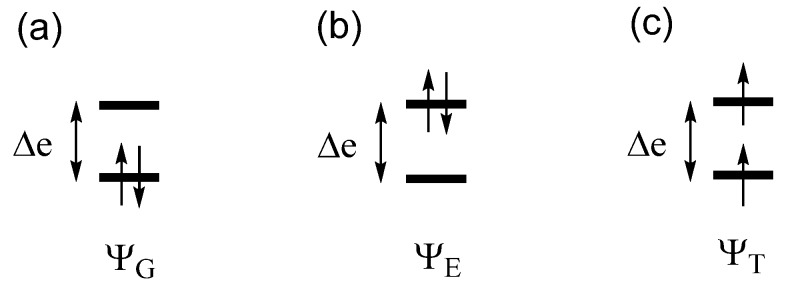
Three configurations critical for determining the energy difference between the singlet and triplet states of a dimer. (**a**,**b**) The two singlet-state configurations. (**c**) The triplet-state configuration.

Let us revisit the relative stabilities of the low-spin and high-spin states of a dimer, discussed above. The low-spin state Ψ_G_ with the bonding level ψ_1_ doubly occupied represents a singlet state and indicates that the dimer has a single bond. The high-spin state Ψ_T_, in which the bonding level ψ_1_ and the antibonding level ψ_2_ are each singly occupied with identical spin, represents a triplet state and indicates that the dimer has neither bonding nor antibonding, in other words, the two electrons are effectively localized at each atomic center of the dimer. The excited configuration Ψ_E_ with the antibonding level ψ_2_ doubly occupied also represents a singlet state (Figure 2). Under the Hamiltonian $\hat{H}$ that generates Ψ_G_, the configuration Ψ_E_ interacts with Ψ_G_, namely, the CI matrix element $\langle \Psi_{G}|\hat{H}|\Psi_{E}\rangle =K_{12}$ is nonzero, if the Hamiltonian $\hat{H}$ includes the electron–electron repulsion term. In the one-electron theory in which $\hat{H}$ neglects electron–electron repulsion, there is no CI. This interaction leads to a mixing between Ψ_G_ and Ψ_E_, leading to a singlet state lower in energy than Ψ_G_, that is, the true ground singlet state is represented by a linear combination of Ψ_G_ and Ψ_E_. When ∆e is small, the weights of Ψ_G_ and Ψ_E_ are comparable in the ground singlet state. If the singlet state is represented by a linear combination of Ψ_G_ and Ψ_E_, it is found [3] that the energy difference between the singlet and the triplet states, ∆E = E_S_ – E_T_ is expressed as

(1) $$\Delta E=2K_{\mathrm{ab}}-\frac{{\left(\Delta e\right)}^{2}}{U}$$

where K_ab_ is the exchange repulsion between the orbitals χ_a_ and χ_b_ (Figure 1a). Since K_ab_ > 0, the triplet state is more stable than the singlet state (i.e., ∆E > 0) if ∆e = 0, i.e., when the two states ψ_1_ and ψ_2_ are degenerate. This explains the Hund’s rule. Equation (1) shows that the triplet state can be more stable than the singlet state even if the two states are nondegenerate (i.e., ∆e ≠ 0) as long as 2K_ab_ is greater than (∆e)^2^/U. Equation (1) was derived for a dimer in which the AO’s χ_a_ and χ_b_ located at different sites interact weakly (Figure 1a), i.e., when the overlap integral S_ab_ = 〈χ_a_|χ_b_〉 between the AOs χ_a_ and χ_b_ is very small. Nevertheless, Equation (1) is valid even if χ_a_ and χ_b_ refer to MOs of an identical molecule with energy difference ∆e. On the basis of Equation (1), we examine two cases in which the triplet state is more stable than the singlet state, which are discussed below.

**Figure 3:**
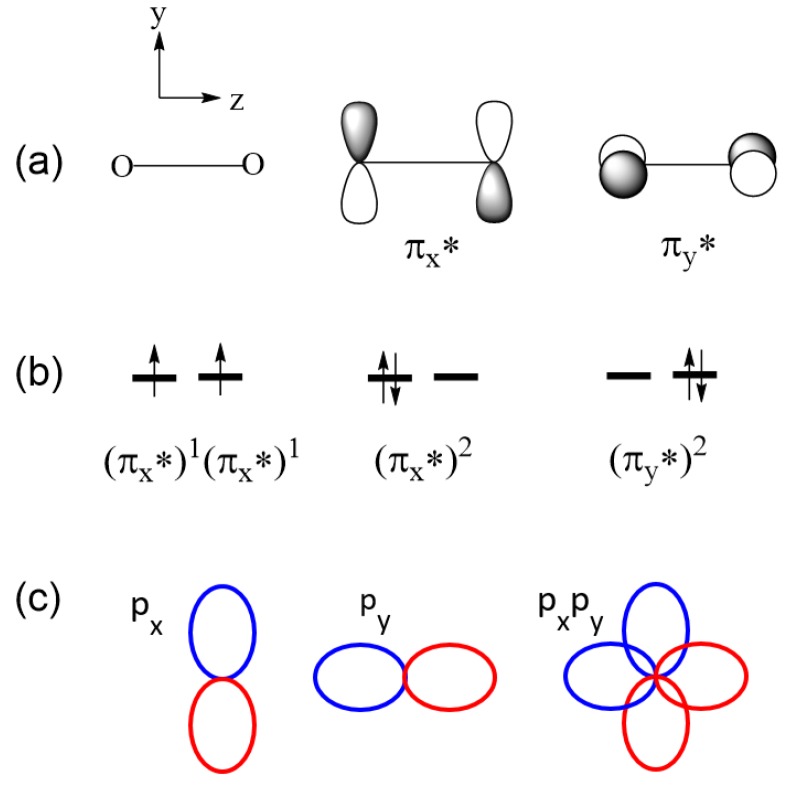
(**a**) Doubly degenerate HOMO of O_2_. (**b**) Three electron configurations describing the triplet and singlet states of O_2_. (**c**) The overlap density, p_x_p_y_, resulting from the p_x_ and p_y_ orbitals at one oxygen site of O_2_. The positive and negative lobes of each p orbital are represented by ellipses of different colors.

The HOMO of triplet oxygen O_2_ consists of two degenerate pi-antibonding MOs, π_x_* and π_y_* (Figure 3a), and there are two electrons to occupy them. The triplet state is represented by the configuration (π_x_*)^1^(π_y_*)^1^, and the singlet state by the linear combination of the two configurations (π_x_*)^2^ and (π_y_*)^2^ (Figure 3b). ∆e = 0 between π_x_* and π_y_* because they are degenerate, so Hund’s rule predicts that the configuration (π_x_*)^1^(π_y_*)^1^ is the ground state. To verify this prediction using Equation (1), we note that the exchange repulsion between π_x_* and π_y_* (corresponding to χ_a_ and χ_b_ of Equation (1), respectively) is nonzero. In general, the exchange repulsion K_ab_ between orbitals χ_a_ and χ_b_ arises from the overlap density $\rho_{\mathrm{ab}}=\chi_{a}\chi_{b}$, namely, K_ab_ is nonzero if $\rho_{ab}$ does not vanish. The overlap density π_x_*π_y_* between π_x_* and π_y_* involves, at each oxygen atom, the overlap density p_x_p_y_ between the p_x_ and p_y_ AOs, the constituents of π_x_* and π_y_*, respectively (Figure 3a). As depicted in Figure 3c, the overlap density p_x_p_y_ has nonzero regions, so the exchange repulsion between π_x_* and π_y_* is nonzero. Thus, the triplet state is more stable than the singlet state in O_2_ (by 22.6 kcal/mol) [4].

**Figure 4:**
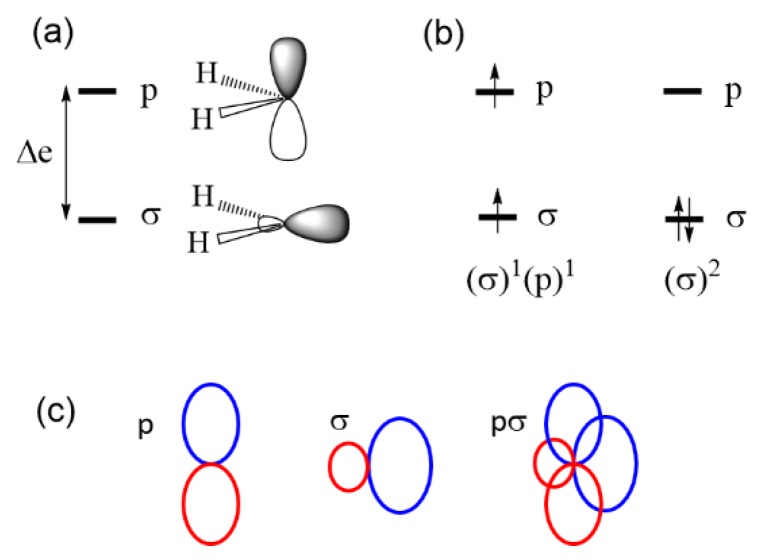
(**a**) Two MOs of carbine CH_2_ determining the triplet and singlet states. (**b**) The triplet and singlet electron configurations of CH_2_. (**c**) The overlap density, pσ, resulting from the σ and p orbitals of CH_2_. The positive and negative lobes of the p and σ orbitals are represented by ellipses of different colors.

Now consider the singlet and triplet states of carbene CH_2_. The two MOs of CH_2_ that determine its triplet and singlet states are essentially the sp^2^-type orbital σ and the p orbital depicted in Figure 4a. The triplet state is represented by the configuration (σ)^1^(p)^1^, and the singlet state by the configurations (σ)^2^ (Figure 4b). The energy difference ∆e between σ and p is nonzero, so the Aufbau principle predicts that the singlet state is the ground state in disagreement with the experiment. Since the σ level is essentially a sp^2^-type hybrid orbital, the overlap density between σ and p becomes substantial (Figure 4c), and so is the associated exchange repulsion. This accounts for why the triplet state is more stable than the singlet state in carbene CH_2_ (by 9 kcal/mol) [5] despite the fact that ∆e is nonzero. 

In short, Equation (1) extends Hund’s rule to cases involving nondegenerate levels. When ∆e is small, one needs to consider the possibility that the predictions of the Aufbau principle can be incorrect.

### 2.3. Limits of the One-Electron Picture

**Figure 5:**
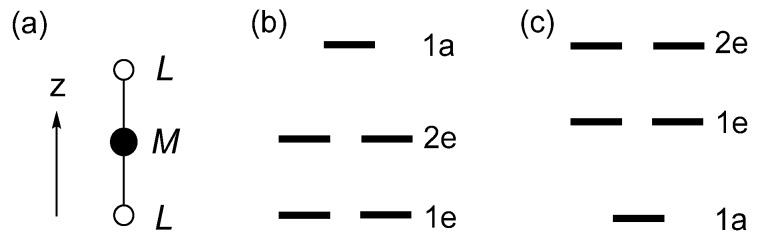
(**a**) Linear *ML*_2_ molecule, where *M* is a transition metal, and *L* a main group ligand. (**b**) The d-state split pattern of 1e < 2e < 1a, predicted by the crystal-field theory (CFT). (**c**) The d-state split pattern of 1a < 1e < 2e assumed to rationalize the uniaxial magnetism of (Fe*L*_2_)^−^ and Co*L*_2_.

**Figure 6:**
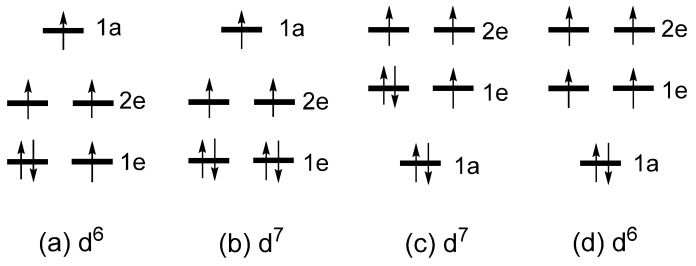
(**a**, **b**) The high-spin d^6^ and d^7^ configurations of *ML*_2_ based on the d-state split patter of 1e < 2e < 1a. (**c**, **d**) The high-spin d^6^ and d^7^ configurations of *ML*_2_ based on the d-state split pattern of 1a < 1e < 2e.

Let us consider a linear molecule Fe*L*_2_ containing a high-spin Fe^2+^ (d^6^, S = 2) ion, where the ligand *L* is bulky, for example, *L* = C[Si(CH_3_)_3_]_3_. With the local *z*-axis of Fe*L*_2_ taken along the axis of its rotational symmetry, i.e., along the *L*-Fe-*L* direction (Figure 5a), the five d-states, xy, x^2^-y^2^, xz, yz and 3z^2^-r^2^, of Fe*L*_2_ are grouped into the 1a, 1e and 2e sets [6,7],

1a = 3z^2^ − r^2^ = |2, 0〉 1e = (xy, x^2^-y^2^) = (|2, 2〉, |2, -2〉) 2e = (xz, yz) = (|2, 1〉, |2, -1〉)(2)

which are based on the magnetic quantum numbers *l*_z_ of the spherical harmonics |*l*, *l*_z_〉 (*l* = 2, *l*_z_ = ±2, ±1, 0) describing the angular properties of the d-orbitals. For simplicity, the term “d-states” are used to indicate the d-block level of Fe*L*_2_ in which the d-orbitals of Fe are combined out-of-phase with s/p orbitals of the ligands *L*. The crystal-field theory (CFT) predicts that the d-states of a transition-metal cation at a linear coordination site are split as 1e < 2e < 1a (Figure 5b), so that the ground-state electron configuration of Fe*L*_2_ containing a Fe^2+^ (d^6^, S = 2) ion is given by (1e)^3^(2e)^2^(1a)^1^ (Figure 6a). Consequently, the degenerate level 1e becomes unevenly occupied. A magnetic system with such an electronic configuration is predicted to have a nonzero magnetic moment only in one direction, i.e., along the *z*-axis. Such a magnetic system is said to be uniaxial [8]. As expected, Fe*L*_2_ is found to be uniaxial [9]. Note that a system with an unevenly occupied degenerate level is susceptible to a Jahn–Teller distortion, which lowers its symmetry by lifting the degeneracy that led to the unevenly occupied degenerate level. Thus, uniaxial magnetism is observed only when the associated Jahn–Teller distortion is prevented. In Fe*L*_2_, the Jahn–Teller instability is blocked by the bulky ligand *L* = C[Si(CH_3_)_3_]_3_ surrounding the Fe^2+^ ion [9].

Linear (Fe*L*_2_)^−^ units containing a Fe^+^ (d^7^, S = 3/2) ion, found in [K(crypt-222)](Fe*L*_2_), exhibit uniaxial magnetism [10]. Linear Co*L*_2_, where *L* = C(SiMe_2_ONaPh)_3_, contains a Co^2+^ (d^7^, S = 3/2) ion. The latter is isoelectronic with Fe^+^ and exhibits uniaxial magnetism [11]. In terms of the d-state split pattern of 1e < 2e < 1a explaining the uniaxial magnetism of Fe*L*_2_, the ground state electron configuration of (Fe*L*_2_)^−^ and Co*L*_2_ would be (1e)^4^(2e)^2^(1a)^1^ (Figure 6b). The latter fails to explain the uniaxial magnetism of (Fe*L*_2_)^−^ and Co*L*_2_ because it has no unevenly occupied degenerate level. To explain the uniaxial magnetism of linear (Fe*L*_2_)^−^ and linear Co*L*_2_ using the Aufbau principle, it was necessary to assume that the d-state split pattern is 1a < 1e < 2e (Figure 5c) [10,11] because the resulting configuration (1a)^2^(1e)^3^(2e)^2^ has an unevenly occupied degenerate level (Figure 6c). However, this d-state split pattern cannot explain the uniaxial magnetism of linear Fe*L*_2_, because the resulting configuration (1a)^2^(1e)^2^(2e)^2^ for the Fe^2+^ (d^6^) ion does not have an unevenly occupied degenerate level (Figure 6d). Thus, we arrive at an impasse of the one-electron picture, as the d-state split pattern needed to explain the uniaxial magnetism of a high-spin d^6^ ion (d^7^ ion) does not explain that of the high-spin d^7^ ion (d^6^ ion) [6,7]. In terms of the CFT, the d-state split pattern of any transition metal cation at a linear coordination site is given by 1e < 2e < 1a. Thus, the Aufbau principle combined with the CFT fails to explain that of the high-spin d^7^ ion of linear (Fe*L*_2_)^−^ and linear Co*L*_2_. 

**Figure 7:**
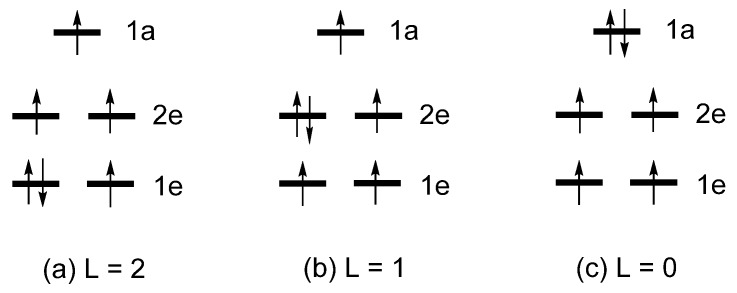
Three electron configurations of Fe*L*_2_ based on the d-state split pattern of 1e < 2e < 1a: (**a**) L = 2, (**b**) L = 1 and (**c**) L = 0 configurations..

**Figure 8:**
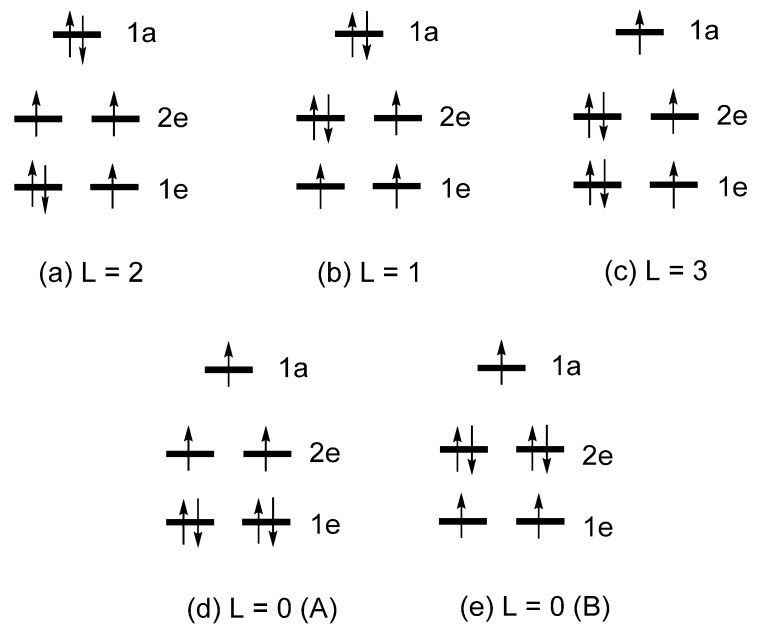
Five electron configurations of (Fe*L*_2_)^−^ and Co*L*_2_ based on the d-state split pattern of 1e < 2e < 1a: (**a**) L = 2, (**b**) L = 1, (**c**) L = 3, (**d**) L = 0 (A), and (**e**) L = 0 (B) configurations.

As already mentioned, the total energies of the various electron configurations of linear Fe*L*_2_, (Fe*L*_2_)^−^ or Co*L*_2_ are determined by the occupation of their split d-states, as well as by the Coulomb and exchange repulsions between the occupied d-states. Figure 7 depicts several electron configurations of Fe*L*_2_, and Figure 8 those of (Fe*L*_2_)^−^ or Co*L*_2_, where each electron configuration is classified by the value of L = $\sum_{i}L_{z}(i)$, where *l*_z_(i) refers to the *l*_z_ value of the occupied d-state i. The relative total energies of these configurations were examined in terms of first principles density functional theory (DFT) including extensive configuration interaction (CI) calculations [6]. These CI calculations for Fe*L*_2_ show that the three states (Figure 7) increase their energies in the order, L = 2 < L = 1 < L = 0, which is exactly what the Aufbau principle predicts based on the d-state split pattern, 1e < 2e < 1a. The CI calculations for (Fe*L*_2_)^−^ and Co*L*_2_ show that the five states (Figure 8) increase their energies in the order:

For (Fe*L*_2_)^−^: L = 2 < L = 1 < L = 3 < L = 0 (A) < L = 0 (B)

For Co*L*_2_: L = 2 < L = 3 < L = 0 (A) < L = 0 (B) < L = 1.

**Figure 9:**
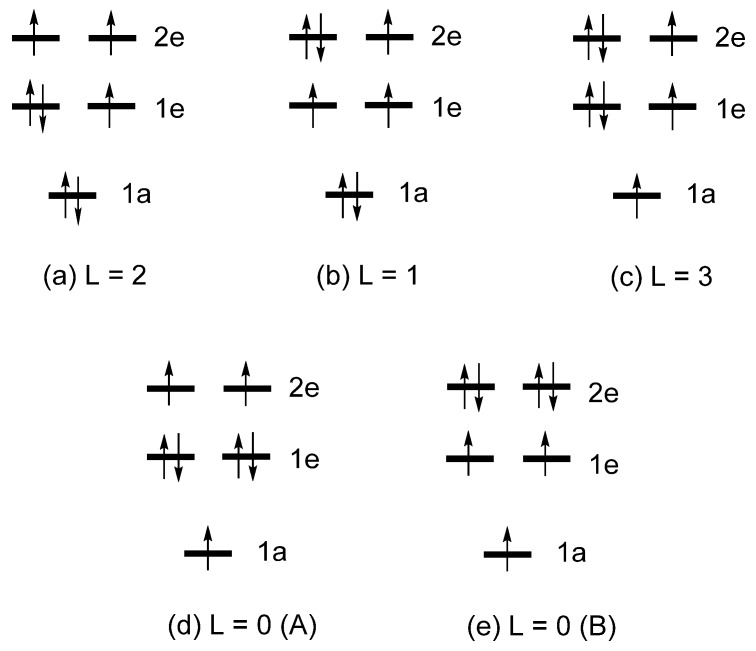
Five electron configurations of (Fe*L*_2_)^−^ and Co*L*_2_ based on the d-state split pattern of 1a < 1e < 2e: (**a**) L = 2, (**b**) L = 1, (**c**) L = 3, (**d**) L = 0 (A), and (**e**) L = 0 (B) configurations.

With the L = 2 state as the ground state, the uniaxial magnetism of both (Fe*L*_2_)^−^ and Co*L*_2_ is explained by the CI calculations. In contrast to this conclusion based on highly extensive CI calculations [6], a computational study based on CASSCF calculations [12] reported that the ground state of (Co*L*_2_)^0^ is the L = 3 state, but details of the CSASCF calculations were not reported. As already pointed out, the trends in the relative energies obtained by the CI calculations are not reproduced by the Aufbau principle using the d-state split pattern, 1e < 2e < 1a. If the Aufbau principle were to be combined with the d-state split pattern of 1a < 1e < 2e, the following relative stabilities can be deduced from Figure 9: 

L = 2 < L = 1 < L = 3, and L = 2 < L = 0 (A) < L = 0 (B).

In short, only the ground state (i.e., the L = 2 state) correctly predicted by the Aufbau principle when combined with the d-state split pattern of 1a < 1e < 2e. As discussed above, the correct ground state for a linear *ML*_2_ system containing a high-spin d^7^ ion is not correctly predicted by the Aufbau principle. It is important to be aware that, though useful as a conceptual framework for discussion, the Aufbau principle can lead to such failures. 

## 3. Effect of Onsite Repulsion on the relative Stabilities of Metallic and Magnetic Insulating States 

### 3.1. One-Electron Approximation

**Figure 10:**
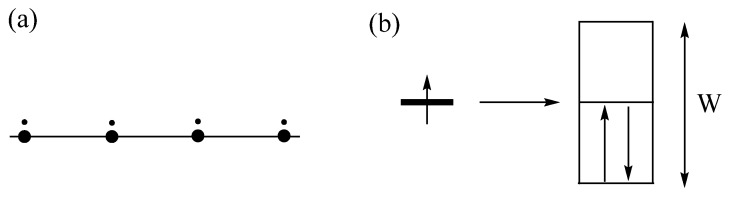
(**a**) A chain made up of identical atoms with one electron and one orbital. (**b**) The formation of an energy band of the chain (right) from the energy level of an atom (left). With one electron per site, each level of the lower-half of the band is doubly occupied in the one-electron picture.

**Figure 11:**
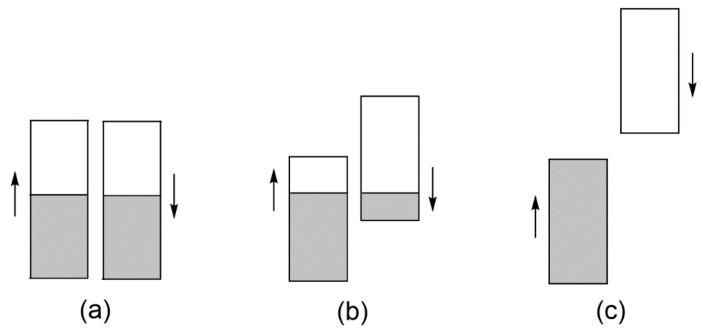
The description of a band in terms of up-spin and down-spin subbands: (**a**) When the up-spin and down-spin bands are not spin-polarized, leading to a nonmagnetic metallic state. (**b**) When the up-spin and down-spin bands are partially spin-polarized, leading to a magnetic metallic state. (**c**) When the up-spin and down-spin bands are completely spin-polarized, leading to a magnetic insulating state.

The electronic structure of a molecule is described by discrete energy levels, and that of an extended solid by energy bands. For simplicity, consider a chain made up of identical atoms with one electron and one orbital per site (Figure 10a). The energy levels allowed for this chain span from the most bonding to the most antibonding levels (Figure 10b) leading to a band of width W ≈ 4|*t*|, where *t* is the hopping integral between the nearest-neighbor sites. With one electron per site, each of the lower half band levels can be doubly occupied so that there is no energy gap between the highest-occupied and the lowest-unoccupied band levels. Thus, the resulting half-occupied band represents a metallic state. In Figure 10b, each allowed level is regarded as accommodating two electrons of opposite spins. However, it is more convenient to think that each allowed level consists of up-spin and down-spin sublevels, which are degenerate in the non-spin-polarized description of electronic structures. In the latter description, the partially occupied band depicted in Figure 10b is identical to that in Figure 11a, and there is no net magnetic moment at each atomic site. In the spin-polarized description of electronic structures, the up-spin and down-spin sublevels are allowed to differ in their spatial orbitals, and hence in their energies. This will be discussed further in the next section. 

### 3.2. Magnetic Insulating State Induced by Onsite Repulsion

When the up-spin and down-spin states of discrete molecules or extended solids become spin-polarized, more up-spin electrons occur than down-spin electrons (by convention). One outcome of the spin polarization is that both up-spin and down-spin bands can become partially occupied, as depicted in Figure 11b. This represents a metallic state in which each atomic site has nonzero magnetic moment, namely, a magnetic metallic state. When the spin polarization is strong, the up-spin and down-spin bands are completely separated so that there is an energy gap between the highest-occupied and lowest-unoccupied energy levels (Figure 11c), and the resulting state becomes a magnetic insulating state. In the metallic state (Figure 11a), each occupied state has two electrons so that the metallic state has onsite repulsion. In the magnetic insulating state (Figure 11c), all occupied states each have one electron of identical spin so that there is no onsite repulsion. Thus, when the width of the band W is greater than the onsite repulsion (i.e., when W > U), the metallic state becomes more stable than the magnetic insulating state, but the opposite is the case if W < U [2,13]. Magnetic insulators arising from the spin polarization induced by onsite repulsion are known as Mott insulators. According to the Aufbau principle, the nonmagnetic metallic state would be more stable than either the magnetic metallic state or the magnetic insulating state. The spin polarization leading to the latter two states takes place to reduce the extent of electron repulsion between occupied states.

### 3.3. Need to Consider beyond Onsite Repulsion

In a given group, the 3d-orbital is more contracted than the 4d orbital, which is in turn more contracted than the 5d orbital. Thus, the onsite repulsion associated with the d-orbitals is strongest for the 3d transition metal element. This is the reason why a greater number of magnetic insulators are found among the compounds of 3d transition-metal elements than among those of 4d and 5d transition-metal elements. Another factor affecting the magnitude of U of a given transition metal atom *M* is the charge of the atom. The d-orbitals of *M* are more contracted in the cation than in the neutral state, so the onsite repulsion U should be greater for the cation and should increase with an increase in the positive charge on the cation. Thus, one might consider it to be unlikely that a compound of a 5d transition-metal cation with a partially occupied band becomes a magnetic insulator. 

**Figure 12:**
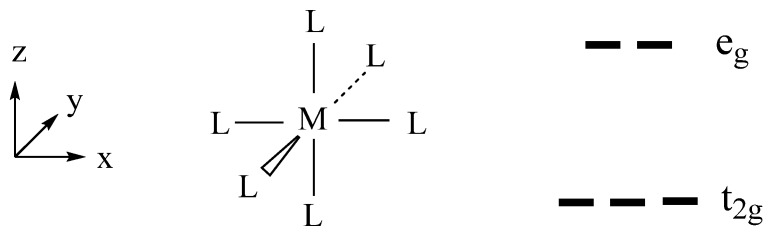
Transition-metal complex ML_6_ octahedron and its d-orbital split pattern.

**Figure 13:**
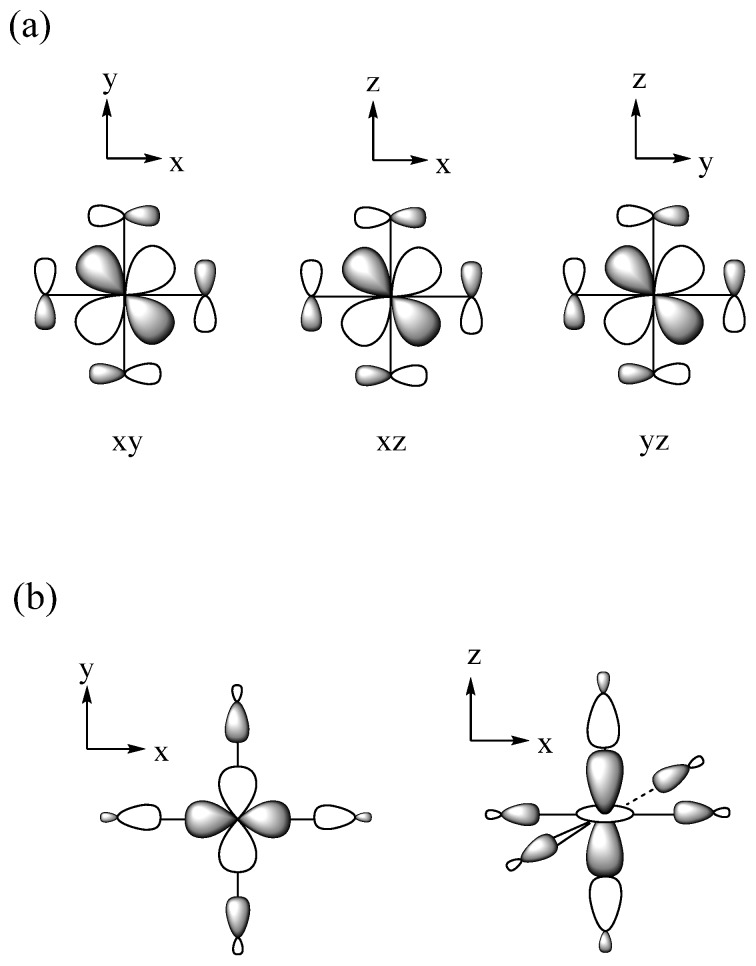
(**a**) The orbital characters of the t_2g_-block states of a ML_6_ octahedron. (**b**) The orbital characters of the e_g_-block states of a ML_6_ octahedron.

**Figure 14:**
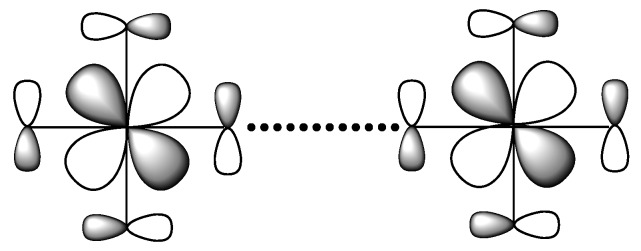
Interaction between two adjacent t_2g_-states in Ba_2_NaOsO_6_.

**Figure 15:**
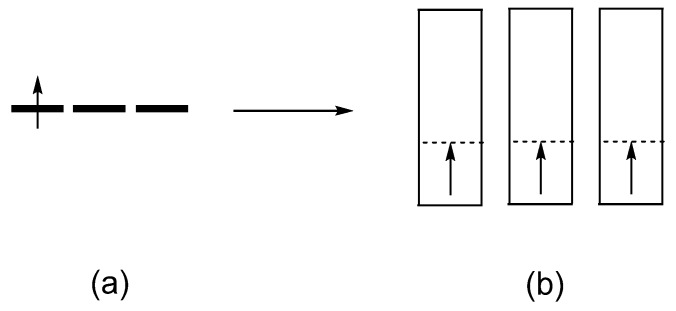
The formation of the t_2g_-block bands of Ba_2_NaOsO_6_ from the t_2g_-level of the individual OsO_6_ octahedra: (**a**) The t_2g_-level of an OsO_6_ octahedron. (**b**) The t_2g_-block bands of Ba_2_NaOsO_6_. With one electron to occupy the three degenerate up-spin bands, one obtains a magnetic metallic state.

However, this reasoning is not necessarily correct. For example, consider Ba_2_NaOsO_6_, consisting of OsO_6_ octahedra containing Os^7+^ (d^1^, S = 1/2) cations. The d-states of each Os^7+^ (d^1^, S = 1/2) cation are split into the t_2g_ and e_g_ states (Figure 12). With the p-orbitals of the surrounding ligands, the metal d-orbital makes pi-antibonding in the t_2g_ states, and sigma-antibonding in the e_g_ states (Figure 13). In this double-perovskite Ba_2_NaOsO_6_ [14], each OsO_6_ octahedron is connected to its neighboring six OsO_6_ octahedra by the Os-O⋅⋅⋅Na^+^⋅⋅⋅O-Os bridges in three orthogonal directions. Since the O⋅⋅⋅Na^+^⋅⋅⋅O bridges have a long the O⋅⋅⋅O contact distance (Figure 14), the t_2g_-states of each OsO_6_ octahedron interact very weakly with those of its adjacent OsO_6_ octahedra. To a first approximation, therefore, the xz, yz and xy states constituting the t_2g_ set of the OsO_6_ octahedra form three very narrow bands. One would expect a small onsite U for the diffuse 5d orbitals, but the U for Os^7+^ may not be negligible because its 5d orbitals might be contracted by the high oxidation state. Since Ba_2_NaOsO_6_ is a magnetic insulator, it is most likely that the W < U condition is fulfilled for Ba_2_NaOsO_6_. This is the condition leading to Mott insulators [13]. However, this condition is not sufficient to provide a magnetic insulating state for Ba_2_NaOsO_6_ because it has one electron to occupy the three degenerate up-spin bands (Figure 15). This leads to a magnetic metallic state for Ba_2_NaOsO_6_ [14], in disagreement with the experimental observation [15]. With any value of U appropriate for Os, it is not possible to introduce a bandgap at the Fermi level. To correct his failure, it is necessary to take into consideration the effects of both SOC and onsite repulsion at the Os^7+^ site [14], as will be discussed in the next section. 

## 4. Effect of Spin-Orbit Coupling

### 4.1. Energy of SOC 

In classical mechanics, the energy of interaction between the spin and orbital moments ($\vec{S}$ and $\vec{L}$, respectively) of an electron in a given atom is written as ξ(r) $\vec{S}$·$\vec{L}$, where the constant ξ(r) decreases with the radius *r* of the electron moving around the nucleus, but increases with the nuclear charge of the atom because the electron density becomes more contracted, hence increasing the relativistic effect. Given the distribution of an electron, the SOC constant 〈ξ〉 of an atom is obtained by integrating ξ(r) over the radial wavefunction R(r) of the atom. Thus, the SOC in an atom of many unpaired electrons with total spin and orbital moments, $\vec{S}$ and $\vec{L}$, respectively, leads to the energy [16],

(3) $$\lambda \vec{S}\cdot \vec{L}$$

where λ = 〈ξ〉/2*S*. For a given oxidation state, the 〈ξ〉 value of an element increases with increasing atomic number while, for a given element, 〈ξ〉 increases with increasing oxidation state. This is so because both factors lead to the contraction of electron density, hence enhancing the relativistic effect. The SOC constant λ is positive when the electron shell containing unpaired electrons is less than half-filled as in V^4+^ (d^1^, S = 1/2). When *λ* > 0, the lowest-energy state is obtained when $\vec{S}$ and $\vec{L}$ are antiparallel with the total moment $\vec{J}$ = $\vec{L}$ − $\vec{S}$. The constant *λ* is negative if the shell is more than half occupied as in Cu^2+^ (d^9^, S = 1/2). When *λ* < 0, the lowest-energy state results when $\vec{S}$ and $\vec{L}$ are parallel with the total moment $\vec{J}$ = $\vec{L}$ + $\vec{S}$. If the shell is half occupied as in high-spin Mn^2+^ (d^5^, S = 5/2), SOC vanishes because $\vec{L}$ = 0 for such an ion. What matters in this discussion is only the relative orientations between $\vec{S}$ and $\vec{L}$. Often, it is more informative to fix the coordinate system describing the orbital angular moment $\vec{L}$ and then ask how the SOC energy $\lambda \vec{S}\cdot \vec{L}$ depends on the orientation of the spin moment $\vec{S}$ with respect to the coordinate system chosen for $\vec{L}$. The importance of the latter approach lies in the fact that a transition-metal cation *M* typically forms an *ML*_n_ (typically, *n* = 2 − 6) complex with the surrounding ligands *L*. Using the coordinate system (x, y, z) for $\vec{L}$ is equivalent to describing the structure of *ML*_n_ using the same coordinate. By convention, the rotational axis of *ML*_n_ is taken as the *z*-axis. This will be discussed in the next section.

### 4.2. Dependence of the SOC Energy on the Spin Orientation

**Figure 16:**
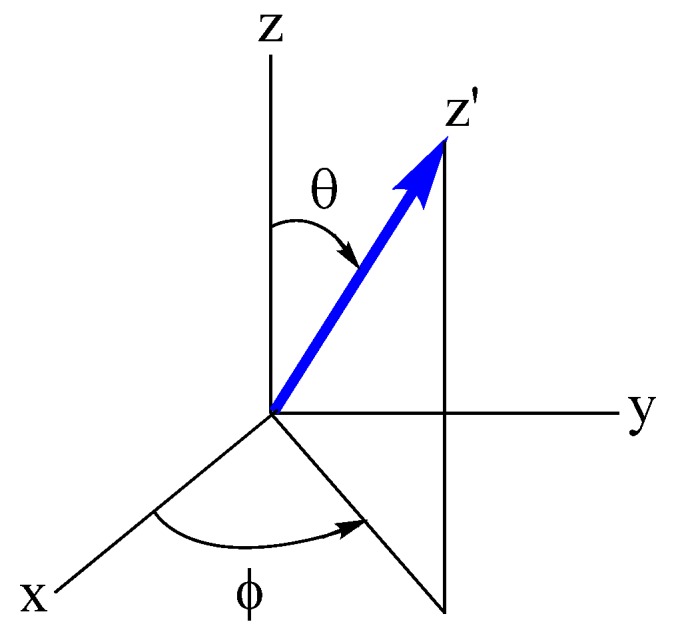
Description of the SOC in terms of two independent coordinate systems; (x, y, z) for $\vec{L}$, and (x′, y′, z′) for $\vec{S}$, where the z′-axis is the spin orientation.

In quantum mechanical description, the SOC energy $\lambda \vec{S}\cdot \vec{L}$ is replaced with $\lambda \hat{S}\cdot \hat{L}$, where $\hat{S}$ and $\hat{L}$ are the spin and orbital momentum operators, respectively. In analyzing the SOC energy $\lambda \hat{S}\cdot \hat{L}$, it is convenient to use two independent coordinate systems (Figure 16); one coordinate system (x, y, z) for $\hat{L}$, and another coordinate system (x′, y′, z′) for $\hat{S}$. Then, the spin orientation is given by the z′-axis, which is defined by the polar angles (θ, φ) with respect to the (x, y, z) coordinate system. An important consequence of using the two independent coordinates is that the SOC energy $\lambda \hat{S}\cdot \hat{L}$ is be rewritten as [16],

(4) $$\lambda \hat{S}\cdot \hat{L}={\hat{H}}_{\mathrm{SOC}}^{0}+{{\hat{H}}^{\prime }}_{\mathrm{SOC}}^{}$$

where the term ${\hat{H}}_{\mathrm{SOC}}^{0}$ allows the SOC interaction only between states of identical spins, and the term ${{\hat{H}}^{\prime }}_{\mathrm{SOC}}^{}$ only between states of opposite spins. The dependence of ${\hat{H}}_{\mathrm{SOC}}^{0}$ and ${{\hat{H}}^{\prime }}_{\mathrm{SOC}}^{}$ on the polar angle θ are expressed as

(5) $${\hat{H}}_{\mathrm{SOC}}^{0}\propto \cos\theta (\alpha {\hat{L}}_{z})+\sin\theta (\beta {\hat{L}}_{+}+\gamma {\hat{L}}_{-})$$

(6) $${{\hat{H}}^{\prime }}_{\mathrm{SOC}}^{}\propto \sin\theta (\alpha^{\prime }{\hat{L}}_{z})+\cos\theta (\beta^{\prime }{\hat{L}}_{+}+\gamma^{\prime }{\hat{L}}_{-})$$

where ${\hat{L}}_{z}$ is the z-component of $\hat{L}$, while ${\hat{L}}_{+}$ and ${\hat{L}}_{-}$ are the raising and lowering operators defined in terms of the x- and y-components of $\hat{L}$. The constants α, β and γ depend on the angle φ, and so do the constants α′, β′ and γ′. When acted on the spherical harmonics $|l,l_{z}\rangle$, these operators lead to the results.

(7) $$\begin{array}{l} {\hat{L}}_{z}|l,l_{z}\rangle \;\propto \;l_{z}|l,l_{z}\rangle \;\\ {\hat{L}}_{\pm }|l,l_{z}\rangle \;\propto \;|l,l_{z}\pm 1\rangle \; \end{array}$$

That is, ${\hat{L}}_{z}$ does not alter the orbital character of $|l,l_{z}\rangle$, but ${\hat{L}}_{+}$ and ${\hat{L}}_{-}$ do. ${\hat{L}}_{+}$ raises the $l_{z}$ value by 1, and ${\hat{L}}_{-}$ lowers the $l_{z}$ value by 1. 

In short, Equations (5) and (6) shows explicitly how the SOC energy $\lambda \vec{S}\cdot \vec{L}$ is affected by the orientation of the spin moment $\vec{S}$ with respect to the coordinate system describing the structure of *ML*_n_. The polar angle θ = 0° means that the spin moment is parallel to the local *z*-axis (||z), i.e., the rotational axis of *ML*_n_, while the angle θ = 90° means that the spin orientation is perpendicular to the *z*-axis (⊥z).

### 4.3. Magnetic Insulating State Induced by Spin-Orbit Coupling and Onsite Repulsion 

**Figure 17:**
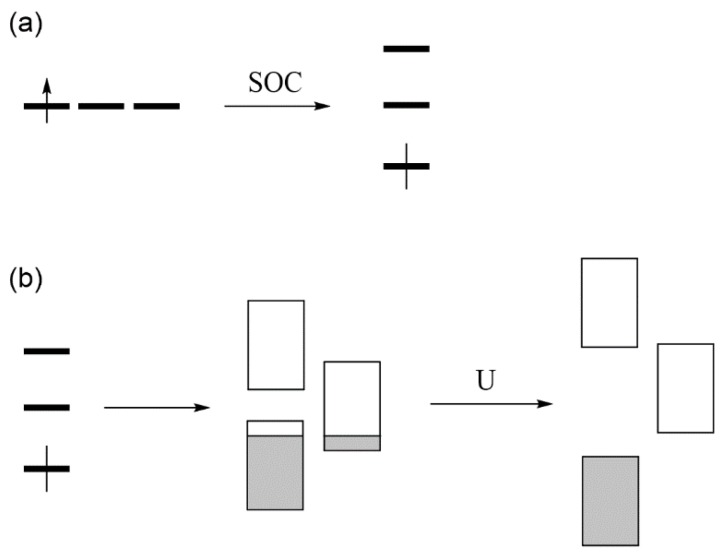
(**a**) The split of the t_2g_ level of an OsO_6_ octahedron by the SOC of Os^7+^. (**b**) The formation of the t_2g_ block bands of Ba_2_NaOsO_6_ from the t_2g_ level split by the SOC, leading to a magnetic metallic state (left), which become magnetic insulating by the spin polarization arising from the on-site repulsion of Os^7+^ (right). Note that, under SOC, there is no distinction between up-spin and down-spin electrons.

We now examine how the xz, yz and xy components of the up-spin t_2g_ state of an OsO_6_ octahedron are affected by the SOC. For this purpose, we note that the angular properties of the xz, yz and xy states are given by

(8) $$\begin{array}{l} \mathrm{xz}\propto |2,-1\rangle -|2,+1\rangle \\ \mathrm{yz}\propto |2,-1\rangle +|2,+1\rangle \\ \mathrm{xy}\propto |2,-2\rangle -|2,+2\rangle \end{array}$$

and the SOC-induced interactions between them are governed by ${\hat{H}}_{\mathrm{SOC}}^{0}$ (Equation (5a)). According to Equation (7), the interaction matrix elements $\langle i|{\hat{H}}_{\mathrm{SOC}}^{0}|J\rangle \;(i,j\;=\;\mathrm{xz},\;\mathrm{yz},\;\mathrm{xy})$ are all nonzero. Namely, the xz, yz and xy states interact under the action of SOC, so that the three t_2g_ levels of an Os^7+^ ion are split into three different states (Figure 17a) [14]. The latter leads to the three slightly overlapping bands (Figure 17b) for Ba_2_NaOsO_6_, still predicting a magnetic metallic state. Once the onsite repulsion is included, the magnetic insulating state is correctly predicted (Figure 17b) [14]. Thus, the consideration of both onsite repulsion and SOC is necessary to explain the magnetic insulating state of Ba_2_NaOsO_6_ [14]. A number of such magnetic insulators have been discovered, which are best described as spin-orbit Mott insulators [17] to distinguish them from Mott insulators. 

### 4.4. Singlet to Triplet Transition

**Figure 18:**
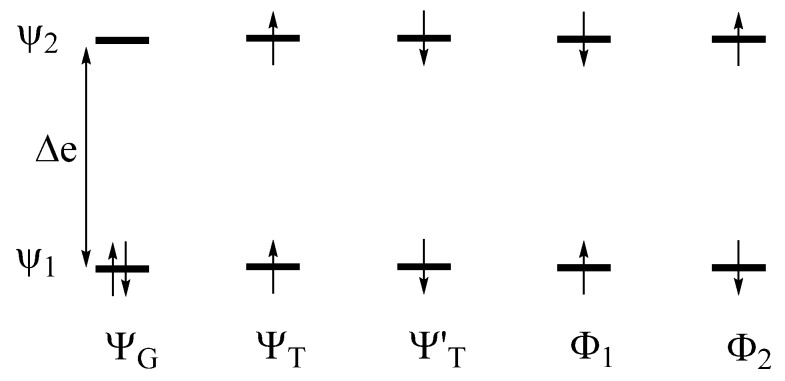
Five configurations leading to the ground and excited singlet states, as well as the excited triplet state. It is assumed that the energy difference ∆e between the two MOs ψ_1_ and ψ_2_ is large, so the ground singlet state is well approximated by Ψ_G_.

**Figure 19:**
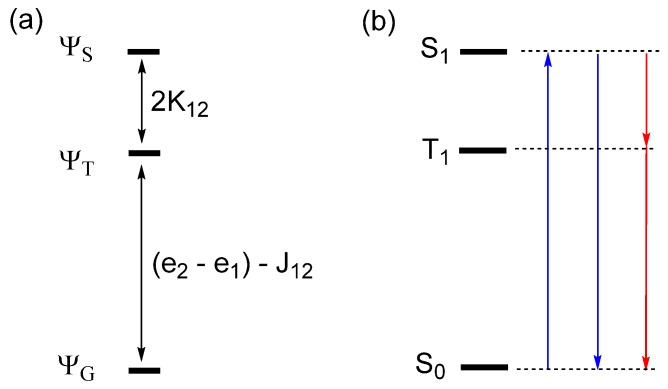
(**a**) The relative energies of the ground state Ψ_G_, the excited singlet state Ψ_S_, and the excited triplet state Ψ_T_, where the MOs ψ_1_ and ψ_2_ used for constructing these configurations are obtained by self-consistent-field (SCF) calculations for Ψ_G_, so e_1_ and e_2_ are the effective orbital energies including electron–electron repulsion. (**b**) The optical transitions associated with the ground and excited singlet and the triplet excited states. In the absence of SOC, the absorption (S_0_ → S_1_) and fluorescence (S_1_ → S_0_) are allowed, but the intersystem crossing (S_1_ → T_1_) and phosphorescence (T_1_ → S_0_) are not.

Let us revisit the two-orbital two-electron problem of Figure 1. If the interaction between the atomic sites is strong, the energy difference ∆e between the MOs is large. Five electron configurations are presented in Figure 18. When ∆e is large, the ground state is well represented by the singlet state Ψ_G_, and the triplet states Ψ_T_ and Ψ′_T_ are higher in energy. The third member of the triplet state, ${\Psi^{\prime \prime }}_{T}$, is expressed as a linear combination of Φ_1_ and Φ_2_. The alternative combination of Φ_1_ and Φ_2_ leads to the excited singlet state $\Psi_{S}$. If the electron repulsions are properly taken into consideration, the

(9) $$\begin{array}{l} {\Psi^{\prime \prime }}_{T}=\frac{\Phi_{1}+\Phi_{2}}{\sqrt{2}}\\ \Psi_{S}=\frac{\Phi_{1}-\Phi_{2}}{\sqrt{2}} \end{array}$$

relative energies of the Ψ_G_, Ψ_T_ and Ψ_S_ states are given as depicted in Figure 19a [18], which shows that their energies increase in the order, Ψ_G_ < Ψ_T_ < Ψ_S_. 

In general, the Ψ_G_ state represents the ground singlet state S_0_ while the Ψ_T_ and Ψ_S_ states represent the first excited triplet and singlet states T_1_ and S_1_, respectively (Figure 19b). Since the singlet and triplet states differ in spin, the transition dipole moment is nonzero between the different singlet states (i.e., S_0_ and S_1_) but vanishes between the singlet and triplet states (e.g., S_0_ and T_1_). Thus, in the absence of SOC, the optical transition is allowed between the singlet states, but not between the singlet and triplet states (Figure 19b). In the presence of SOC, the singlet and triplet states (e.g., S_1_ and T_1_, respectively) interact weakly via the term ${{\hat{H}}^{\prime }}_{\mathrm{SOC}}^{}$ (Equation (6)). As a result, the true excited ‘singlet’ and excited ‘triplet’ states are not pure spin states but are mixed-spin states, namely,

(10) $$\begin{array}{l} {S^{\prime }}_{1}\approx S_{1}+\delta T_{1}\\ {T^{\prime }}_{1}\approx T_{1}+\delta S_{1} \end{array}$$

where δ represents a small mixing coefficient. Consequently, the intersystem crossing occurs between the S_1_ and T_1_ states, and the phosphorescence takes place between the T_1_ and S_0_ states (Figure 19b). 

## 5. Polarizability 

### 5.1. Induced Polarization, Polarizability and Second Harmonic Generation

The dynamic polarizability of a material refers to its ability to generate instantaneous dipoles in response to an external field. With ε_0_ as the electric permittivity of the vacuum, the electric polarization P_i_ (i = x, y, z) of a material induced by the oscillating electric field E_i_ (i = x, y, z) of light is written as,

(11) $$\begin{array}{l} P_{i}(\omega_{1},\;\omega_{2},\cdot \cdot \cdot )=P_{i}^{\left(1\right)}+P_{i}^{\left(2\right)}+\cdot \cdot \cdot \\ =\;\varepsilon_{0}\sum_{J=x,y,z}\chi_{iJ}^{\left(1\right)}(\omega_{1})E_{J}(\omega_{1})+\varepsilon_{0}\sum_{J,K=x,y,z}\chi_{iJK}^{\left(2\right)}(\omega_{1}+\omega_{2},\omega_{1},\omega_{2})E_{J}(\omega_{1})E_{K}(\omega_{2})+\cdot \cdot \cdot \end{array}$$

where $\chi_{iJ}^{\left(1\right)}$ is the linear electric susceptibility, and $\chi_{iJK}^{\left(2\right)}$ is the second order electric susceptibility tensor, or the second harmonic generation (SHG) coefficient tensor when $\omega_{1}$ = $\omega_{2}$. The latter is nonzero only when the system is non-centrosymmetric. Nonlinear optical (NLO) crystals are the key materials for the laser science and technology due to their ability to double the frequency of an incident laser beam through the SHG process. Over the years, a number of studies have been devoted to the understanding of which structural and electronic aspects of NLO materials govern the strengths of their SHG responses. 

For an isotropic system such as an isolated atom, the linear electric susceptibility is isotropic, leading to the atom polarizability

(12) $$\alpha =\frac{P^{\left(1\right)}}{\varepsilon_{0}E}$$

where $P^{\left(1\right)}$ and E are the magnitudes of $P_{i}^{\left(1\right)}$ and E_i_, respectively. In general, the polarizability of an atom becomes larger when the distribution of its valence electron density becomes more diffuse, i.e., when its size becomes larger. To a first approximation, the atoms making up a material are independently polarized so that the total polarizability A_sum_ of the material may be given by the sum [19],

(13) $$A_{SUm}=\sum_{\mu }\alpha_{\mu }^{}$$

where α_μ_ is the polarizability of an atom μ in the material. In the study of piezoelectric crystals, it was shown that the second harmonic nonlinear optical susceptibility can be related to the linear susceptibility as [20],

(14) $$\chi_{iJK}^{\left(2\right)}(2\omega )=\chi_{\mathrm{ii}}^{\left(1\right)}(2\omega )\chi_{JJ}^{\left(1\right)}(\omega )\chi_{KK}^{\left(1\right)}(\omega )\delta_{iJK}^{2\omega }$$

where $\delta_{\mathrm{ijk}}^{2\omega }$ is Miller’s delta parameter. For nonzero SHG coefficients $\chi_{\mathrm{ijk}}^{\left(2\right)}$, and the $\delta_{\mathrm{ijk}}^{2\omega }$ tensor does not deviate much from the mean value for all the crystals investigated despite the fact that their nonlinear susceptibilities vary over orders of magnitude. This finding indicates that an SHG coefficient $\chi_{\mathrm{ijk}}^{\left(2\right)}$ has a multilinear functional dependence on the linear susceptibilities, implying that the second order nonlinear optical property can be understood from the first order property. Miller’s rule implies that a non-centrosymmetric crystal made up of atoms with high polarizability will have a large SHG response.

### 5.2. Polarizabilities of Cations and Anions

**Figure 20:**
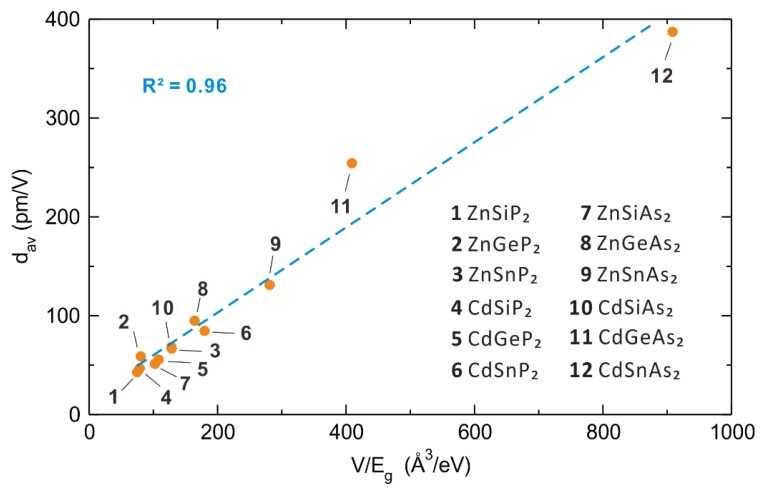
The relationship between SHG (*d*_av_) and *V*/*E*_g_ of the 12 *ABC*_2_ (*A* = Zn, Cd; *B* = Si, Ge; *C* = P, As) chalcopyrites.

In interpreting the properties of a material, it is not the polarizabilities of neutral atoms but those of their cations and anions that are relevant. An atom increases its size when it becomes an anion so that its valence electron distribution becomes more diffuse, hence increasing its polarizability. The opposite occurs when an atom becomes a cation. Calculated atom polarizabilities are known for most neutral atoms [21], but hardly so for their anions and cations. Experimental atom polarizabilities are not known for many neutral atoms, and are known only for a limited few anions and cations. Therefore, it has been a challenging task to estimate the polarizabilities of anions and cations. Very recently, a practical solution to this problem has been found in the study of the 12 *ABC*_2_ (*A* = Zn, Cd; *B* = Si, Ge, Sn; *C* = P, As) chalcopyrites consisting of *A*^2+^, *B*^4+^ and *C*^3−^ ions [18]. The SHG responses of these isostructural NLO compounds increase almost linearly with *V/E*_g_ (Figure 20), where *V* and *E*_g_ are the primitive unit cell volume and the bandgap of a specific *ABC*_2_ compound, respectively. In short, the larger the primitive unit cell volume, the larger the SHG response. This is understandable because the unit cell volume increases when its constituent atoms are large. 

To quantify this point, it is necessary to estimate the polarizabilities of the *A*^2+^, *B*^4+^ and *C*^3−^ ions. In general, the polarizability α_μ_ for an atom μ of radius *r*_μ_ is proportional to its sphere volume (4π/3)*r*_μ_^3^,

α_μ_ ∝ (4π/3)*r*_μ_^3^. (15)

This relationship is expected to be valid even for anions and cations. The polarizabilities of anions and cations can be estimated by employing the following four steps: (a) For the F^−^, O^2−^ and Cl^−^ anions, their ionic polarizabilities are known [22,23,24], and so are their ionic radii [24]. A reasonably good linear relationship exists between the two quantities [19]. (b) This linear relationship can be used to estimate the ionic polarizabilities of various ions from the known ionic radii [25]. (c) When the ionic radii of the ions (e.g., P^3−^ and As^3−^ anions) are unknown, we note for various ions that the polarizabilities derived from the above two steps vary almost linearly with the calculated polarizabilities of their neutral atoms [19]. (d) Finally, this linear relationship is used to estimate the polarizabilities of the ions (e.g., P^3−^ and As^3−^ anions) from those of their neutral atoms (i.e., P and As) [19].

**Figure 21:**
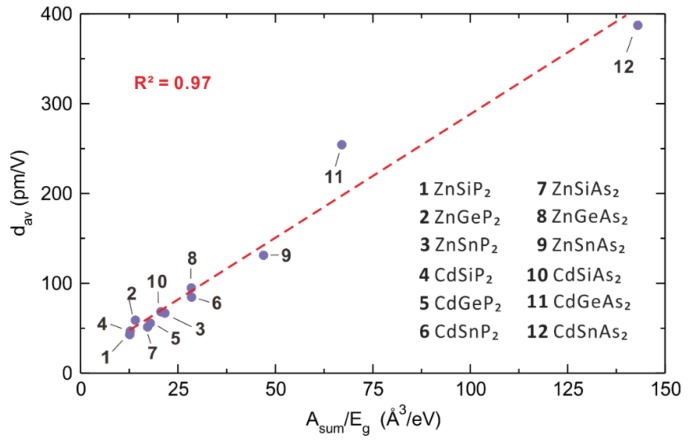
The relationship between SHG (*d*_av_) and *A*_sum_/*E*_g_.

Thus, for each member of the 12 *ABC*_2_ (*A* = Zn, Cd; *B* = Si, Ge, Sn; *C* = P, As) chalcopyrite family, we obtain the total polarizability A_sum_ of a primitive unit cell by summing the individual ion contributions (Equation (13)). The relationship between A_sum_ and *V* is linear, and so is the relationship between the SHG responses and A_sum_/*E*_g_ values (Figure 21). This relationship establishes a clear link between the SHG response and the first order optical response, which can be understood in terms of Miller’s rule. Notice that 12 *ABC*_2_ chalcopyrites are isostructural, so the number of atoms per primitive unit cell is identical. When we compare the SHG responses of non-isostructural NLO compounds, it is necessary to employ the A_sum_/*E*_g_ normalized to an identical number of atoms, for example, A_sum_/(*NE*_g_), where *N* is the number of atoms per unit cell [26].

## 6. Experimental Trends

### 6.1. Ionic Sizes and Oxidation States

**Figure 22:**
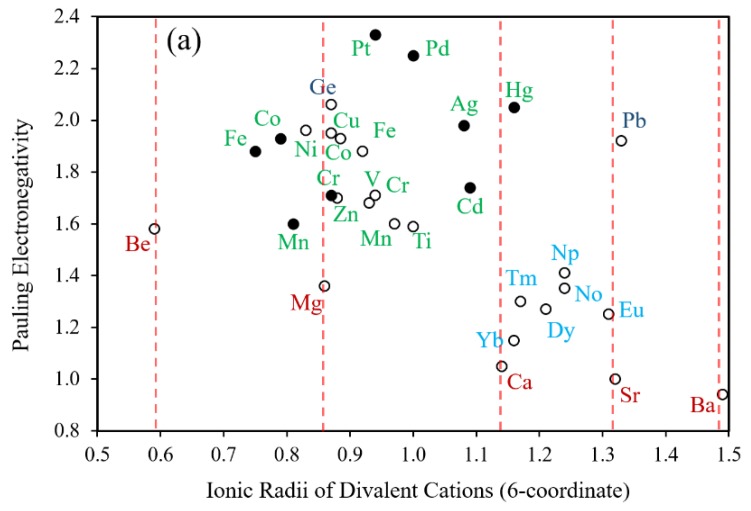

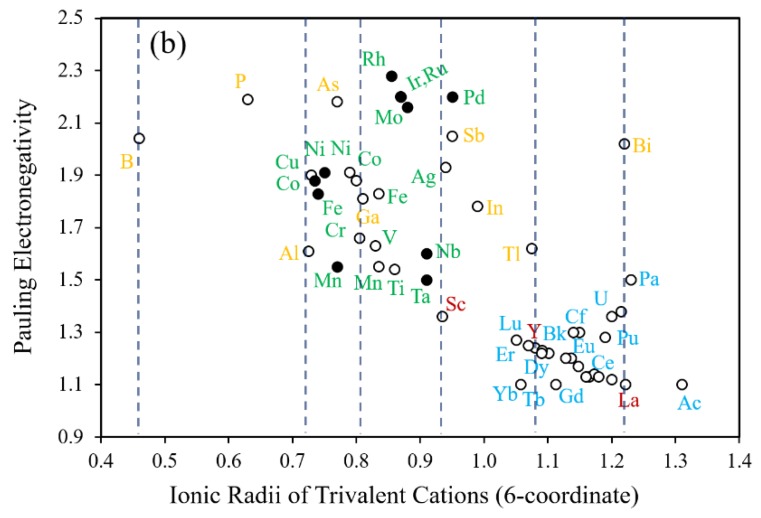
Plots of the crystal radii versus Pauling electronegativity of (**a**) divalent and (**b**) trivalent TM and RE cations, where the low-spin and down-spin cations are represented by shaded and unshaded circles, respectively.

It is well known that the ionic size of an element in a given group increases going down the period (e.g., from Mg^2+^ to Ca^2+^ to Sr^2+^). This allows one to sterically tune the structure of a compound. The ionic sizes and oxidation states of many transition-metal (TM) and rare-earth (RE) elements are relatively similar to those for many main-group elements, rendering them exchangeable within a compound. Figure 22a shows the electronegativity vs. ionic radius plot for various divalent cations at six-coordinate sites, and Figure 22b the corresponding plot for various trivalent cations at six-coordinate sites. Many divalent TM cations have ionic sizes similar to that of Mg^2+^, but they have higher electronegativities and possess d-electrons as valence electrons (Figure 22a). The latter provides added capabilities to tune the bonding, stability and electronic properties. Spanning the interval in ionic size between Ca^2+^ and Sr^2+^ cations are many divalent RE cations. Figure 22b reveals that the sizes of trivalent TM cations lie between those of Al^3+^ and In^3+^ (roughly between those of As^3+^ and Sb^3+^). The sizes of trivalent RE cations lie in the region between those of Tl^3+^ and Bi^3+^ (roughly between those of Y^3+^ and La^3+^).

### 6.2. Onsite Repulsion and d-Electron. Count

In a sense, the onsite repulsion U is a measure of how strongly its valence atomic orbitals are contracted. Thus, the onsite repulsion of an atom μ, U_μ_, would increase with decreasing ionic volume V_μ_ = (4π/3)*r*_μ_^3^, and would increase with increasing oxidation state ζ_μ_. Therefore, the onsite repulsion U_μ_ is expected to increase with the ratio ζ_μ_/V_μ_. Thus, to a first approximation, it may be assumed that

(16) $$U_{\mu }\propto \frac{\zeta_{\mu }}{V_{\mu }}.$$

An important factor affecting the ionic radius of a TM cation *M* at an octahedral site is how the d-states of the *ML*_n_ complex are occupied. The d-states of a *ML*_6_ octahedron are split into t_2g_- and e_g_-states (Figure 12). The t_2g_-states are π-antibonding (Figure 13a), but the e_g_-states are σ-antibonding (Figure 13b), between *M* and *L* so the e_g_-states are more strongly antibonding than are the t_2g_-states. Thus, with respect to the occupation of the t_2g_-states, that of the e_g_-states lengthens the *M*–*L* bond more so that the high-spin states of *ML*_n_ have longer *M–L* bonds than the low-spin *ML*_n_. Effectively, therefore, the cation *M* in the high-spin state has a larger ionic radius, and hence a larger ionic volume. 

**Figure 23:**
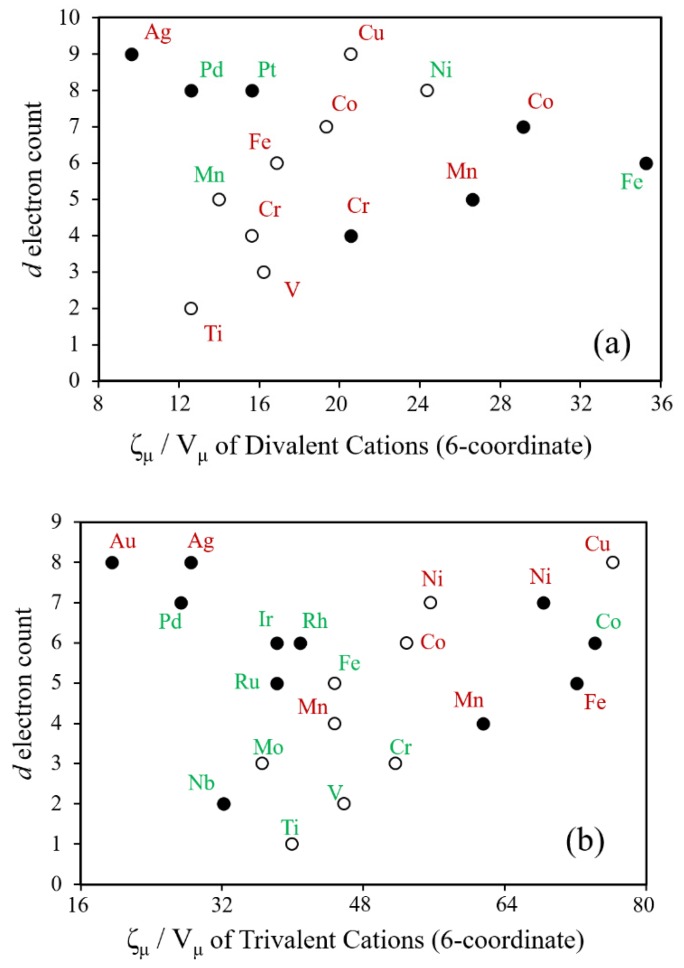
Plots of the d-electron count versus ζ_μ_/V_μ_ for (**a**) divalent and (**b**) trivalent TM cations in an octahedral coordination environment, where the low-spin and high-spin cations are represented by shaded and unshaded circles, respectively. The elements in red are susceptible to a Jahn–Teller distortion or may show uniaxial magnetism.

Shown in Figure 23 are the plots of the d-electron count vs. the ζ_μ_/V_μ_ for various divalent and trivalent TM cations in six-coordinate environments. For both divalent and trivalent TM cations, we find the following: (a) The ζ_μ_/V_μ_ values for low-spin 3d cations increase as the d-electron count increases, reaching a maximum approximately at the d^6^ electron count. This follows the trend that, on going from left to right within a given period, the effective nuclear charge, and hence the electronegativity, increases, thereby contracting the size of the cation. (b) For a given TM cation, the ζ_μ_/V_μ_ value is smaller in the high-spin than in the low-spin state, because the ionic volume becomes larger in the high-spin state due to the fact that the e_g_ state is more antibonding than the t_2g_ state (see Figure 13). (c) The ζ_μ_/V_μ_ values for the 4d and 5d elements are smaller than those of the 3d elements, due to fact that their ionic volumes are larger. These observations are expected from our discussions on the relative stabilities of high-spin and low-spin states (see Section 2.1). (d) The effective onsite repulsion of a trivalent TM cation is greater than the corresponding divalent TM cation, because the trivalent ions are smaller in size than the corresponding divalent cations (see Figure 22). 

Electronic transitions between localized (magnetic) and delocalized (metallic) states can be tuned using the above periodic trends because they can affect whether U > W or U < W. For example, the tendency toward a more metallic/delocalized behavior can be expected by substituting earlier transition metal cations into a compound (e.g., FeO to TiO in rock-salt type structures). In addition, an increase in oxidation state significantly increases the onsite repulsion by a factor of about three (e.g., Ti^2+^ to V^3+^ for the d^2^ electron counts), leading to more localized states. A number of TM cations at octahedral sites are susceptible to a Jahn–Teller distortion, as indicated in Figure 23. Uniaxial magnetism has been observed in the cases when the Jahn–Teller distortion is suppressed, as discussed above. Compounds containing 4d and 5d TM cations show a more metallic behavior than those containing the 3d TM cations because 4d and 5d TM cations possess a smaller onsite repulsion. The exceptional case occurs for spin-orbit Mott magnetic insulators of 5d TM cations, which arise from the combined effect of smaller onsite repulsion and stronger SOC. (see below). 

### 6.3. Onsite Repulsion and Spin Orbit Coupling

**Figure 24:**
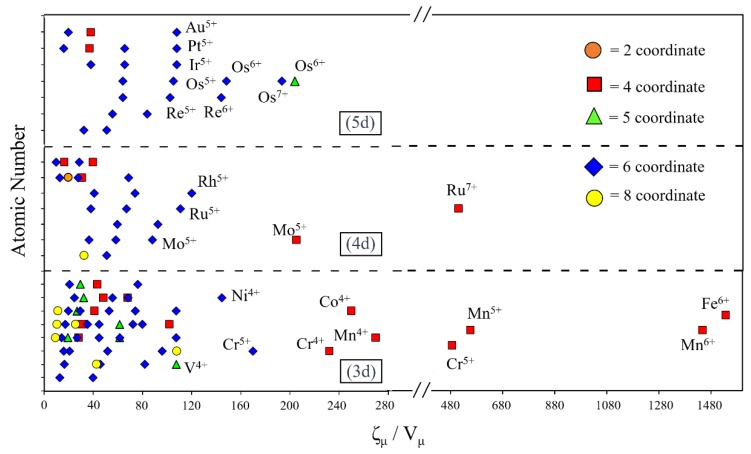
Plots of ζ_μ_/V_μ_ versus the atomic number for the 3d, 4d and 5d TM cations. The coordination numbers of the cations are defined by the legends in the inset.

The SOC of an atom increases with increasing the atomic number Z (i.e., approximately proportional to Z^4^) [27]. To examine how the onsite repulsion of TM cations are correlated with their SOC, we plot the ζ_μ_/V_μ_ values of 3d, 4d and 5d TM cations against their Z values (Figure 24). In analyzing this correlation, we should note that a TM cation *M* with a lower coordination number has a shorter *M*-*L* distance with the ligand *L* because there is less steric crowding among the surrounding ligands *L*. That is, a TM cation *M* with a lower coordination number has a smaller ionic size. It is found from Figure 24 that: (a) TM cations with large ζ_μ_/V_μ_ values possess high oxidation states and low coordination number (e.g., 4-coordinate Fe^6+^ or 4-coordinate Mn^6+^); (b) TM cations with large ζ_μ_/V_μ_ values are found for several 3d cations, a few for 4d cations, and none for 5d cations. There are several factors leading to this trend. First, 3d cations are small in size so their coordination number can become small. This effectively shortens the metal–ligand distance *M–L*, hence effectively reducing the ionic size and increasing the onsite repulsion. Such an effect does not occur for 5d cations because they are large in size, and hence their coordination number cannot become small. Thus, the ζ_μ_/V_μ_ value of 5d cations cannot become large by reducing their coordination number. Second, a high oxidation state is common for 5d cations because their electronegativity is low, which has the effect of increasing the onsite repulsion. The first factor dominates over the second factor (e.g., large ζ_μ_/V_μ_ for 4-coordinate Fe^6+^ vs. small ζ_μ_/V_μ_ for six-coordinate Os^6+^, large ζ_μ_/V_μ_ for four-coordinate Mn^6+^ vs. small ζ_μ_/V_μ_ for six-coordinate Re^6+^). (c) The TM cations with small ζ_μ_/V_μ_ values occur for cations with high coordination numbers (i.e., six- and eight-coordination) largely because their cations are large in size. (d) For 3d, 4d and 5d cations, the ζ_μ_/V_μ_ value are very small for cations with low oxidation states (i.e., below +4, not labeled in Figure 24 for clarity). 

Recent experiments show that SOC effects can start to predominate in the region of Figure 24 containing Ir^5+^, Os^5+^ and Os^7+^ cations in octahedral coordination environments (e.g., Ba_2_NaOsO_6_ discussed in Section 3.3 and Section 4.3 [13,14] as well as other systems, such as (Na/Li)_2_IrO_3_, Sr_2_IrO_4_, NaOsO_3_, and Cd_2_Os_2_O_7_ [26]. Interestingly, this region also shows a close grouping together with Pt^5+^, Re^5+^ and Ir^5+^, suggesting that similar compounds with these cations in octahedral coordination environments should show strong SOC effects. The onsite repulsion can be increased or decreased by using Os^6+^ or W^5+^ cations, respectively, while maintaining a similar SOC effect. To date, however, many of these cations are not known to occur in structures with these combinations of coordination environments and oxidation states. 

## 7. Concluding Remarks

We briefly reviewed how the onsite repulsion, spin–orbit coupling and polarizability of elements and their ions affect the physical properties of molecules and condensed materials. When combined with the energy levels generated by the one-electron theory, the Aufbau principle provides a conceptual tool with which to predict the ground state of molecules and condensed materials, although this approach can fail to provide correct predictions. In most cases, this deficiency of the Aufbau principle can be remedied by introducing onsite repulsion. For magnetic insulators of 5d transition-metal elements, termed spin–orbit Mott insulators, the consideration of both onsite repulsion and SOC is necessary in creating a bandgap. Anions and cations differ in their polarizability from their neutral analogues. Nevertheless, the polarizabilities of ions can be estimated on the basis of their ionic radii and the polarizabilities of their neutral analogues. The onsite repulsions, SOC and polarizabilities of transition metal cations are interrelated, and this interrelationship can be used to tune these parameters experimentally.
